# From climate to crop: Unveiling the impact of agro-climate dataset on rice yield in Cotabato Province

**DOI:** 10.1016/j.dib.2023.109754

**Published:** 2023-11-05

**Authors:** Reymark D. Delena, Marivic S. Tangkeko, Joseph C. Sieras

**Affiliations:** a Department of Information Sciences, College of Information and Computing Sciences, Mindanao State University, Marawi City, Philippines; b Department of Information Technology, College of Computer Studies, De La Salle University, Manila City, Philippines

**Keywords:** Cotabato Province, Irrigated and rainfed rice production, Analytical dashboard, Climate dataset, Data Visualization

## Abstract

This data article presents a dataset that analyzes the trends in climatic factors and rice yield in Cotabato Province, a key contributor to the country's rice output. The dataset was collected from the Office of the Provincial Agriculturist and NASA's POWER Prediction Of Worldwide Energy Resources (POWER) dataset agro-climate dataset from 2007 to 2021. Moreover, the data was processed using Extract, Transform, and Loading (ETL) method, and multivariate linear regression analysis was conducted to identify the agro-climates that significantly influence the production of irrigated and rainfed rice. Further, the explanatory factors that significantly influence the production of rice were determined and presented in an Analytical Dashboard. The dataset has great reuse potential for predictive analytics research at the municipal level, which can provide more detailed insights into the agro-climates of different municipalities in Cotabato Province. Moreover, the dataset can also be used to distribute different varieties of rice that can withstand the effects of climate change to the municipalities of Cotabato. Overall, this dataset provides valuable insights into the relationship between agro-climate and rice production in Cotabato Province and can inform future decision-making and resource allocation in the region.

Specifications Table

**Table:** 

Subject	Business, Management and decision sciences, Environmental Science, Agricultural Science
Specific subject area	The analysis and visualization of the relationship between agro-climate and rice production in Cotabato Province, including the identification of influential factors and creation of an analytical dashboard for decision-making.
Data format	Raw, Analyzed, Filtered
Type of data	Table, Image, Chart, Graph, Figure
Data collection	Rice yield data from the Office of the Provincial Agriculturist, and NASA's POWER (Prediction Of Worldwide Energy Resources) project agro-climate dataset were collected and processed using ETL from 2007 to 2021.
Data source location	The institution, known as the Office of the Provincial Agriculturist, is situated in Cotabato Province, which is a region located in the southern part of the Philippines.
Data accessibility	Repository name: Mendeley Data Data identification number: 10.17632/5tp2p84mzc.4 Direct URL to data: https://data.mendeley.com/datasets/5tp2p84mzc/4
Related research article	Delena, R. D., Tangkeko, M. S., Ampuan, A. D., & Sieras, J. C. (2023). ARP Cotabato: Exploring seasonal climate and rice production in Cotabato province through advanced data visualization and rapid analytics. Software Impacts, 17, 100546. https://doi.org/10.1016/j.simpa.2023.100546

## 1 Value of the Data

• Researchers studying the intricate relationship between agro-climate conditions and rice production in Cotabato Province can rely on this data. They can identify patterns and correlations by analyzing historical records, gaining crucial insights into how climate impacts rice cultivation. Such knowledge is essential for devising strategies to enhance agricultural productivity in the region.
• This dataset is a valuable resource for local and regional decision-makers. It provides essential information for making informed choices regarding resource allocation, agricultural policies, and interventions. These decisions have far-reaching consequences, impacting the livelihoods of farmers and the overall economic stability of the area.
• The availability of this data also opens doors for future research in predictive analytics, especially at the municipal level. Predictive models can be developed to anticipate the effects of changing climate conditions on rice production, allowing for proactive measures and adaptation strategies.
• Researchers from various fields, including agriculture and climate science, can benefit from this dataset. It supports interdisciplinary research efforts aimed at addressing complex challenges related to food security, climate resilience, and sustainable agriculture.

## 2 Data Description

The data presented pertains to the agricultural dataset of Cotabato, Philippines from 2007 to 2021. The agricultural dataset included information on crop-related and agro-climate data. We conducted several critical data processing steps to enhance their utility and relevance to our research [1]. This included data cleaning, quality control measures, and the computation of annual means. For instance, by processing the data in this manner, we transformed the primary datasets into a more refined, analytically useful form. This allowed us to draw meaningful insights and conduct a thorough analysis. Our value addition through data processing forms the foundation for the development of an agro-climate and rice production analytical dashboard [2], and this dataset is accessible in [3].

In the agricultural dataset, the crop-related data contained two (2) crop types: the irrigated and rainfed. Also, in the agro-climate data, there are five (5) climatic conditions included such as precipitation, radiation, soil moisture, humidity, and temperature.

### 2.1 Crop-Related Data

This data presents crop-related information specific to Cotabato Province, organized by year and municipality. It primarily focuses on rice production and planting area, breaking into irrigated and rainfed categories. All the parameters used to describe the data are listed in Table 1.

**Table 1:** Key variables measured, short name, and units.

Variable	Sub-variable	Description	Unit
Rice Production	Irrigated	This variable quantifies the volume of rice harvested from fields where controlled irrigation systems are employed in the specified municipality and year. It is measured in metric tons (t) and represents rice cultivation with water system.	t
	Rainfed	This variable measures the quantity of rice obtained from fields that rely primarily on natural rainfall for irrigation in the specified municipality and year. It is also measured in metric tons (t) and reflects rice farming without the use of controlled irrigation systems, depending on seasonal precipitation.	t
Planting Area	Irrigated	This variable signifies the total land area specifically allocated for rice cultivation under controlled irrigation systems in hectares (h) within the specified municipality and year. It quantifies the extent of land dedicated to rice farming with water supply management.	h
	Rainfed	This variable denotes the total land area designated for rice cultivation that predominantly relies on natural rainfall for irrigation in hectares (h) within the specified municipality and year. It quantifies the land area used for rice farming without the use of controlled irrigation systems, dependent on seasonal precipitation.	h

**Fig. 1:**
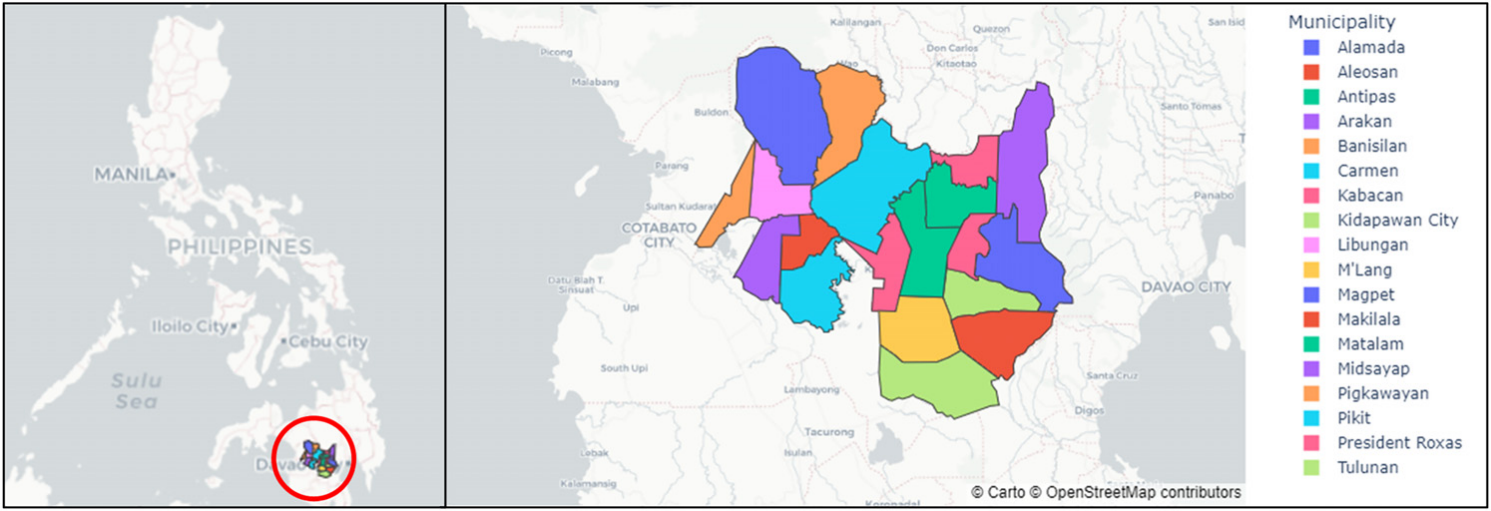
Map of the Cotabato Province in the Philippines with location used in simulation.

**Fig. 2:**
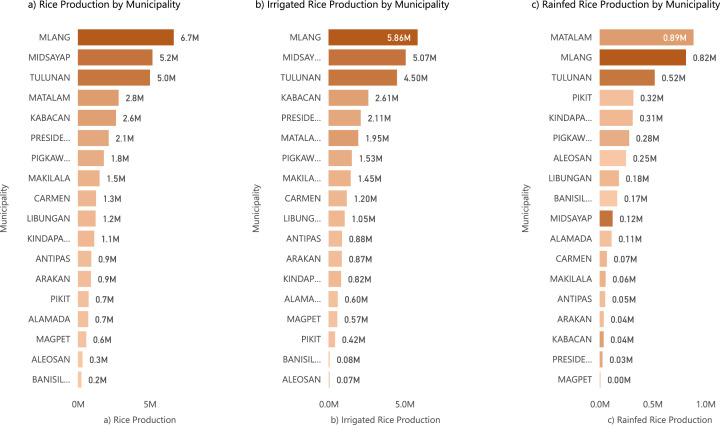
Rice production data of the municipalities in Cotabato Province from 2007 to 2021 from (a) the average rice production of per municipalities; (b) the irrigated production performance; and (c) the rainfed production performance of each municipality.

The dataset was carried out in 18 different municipalities in the province of Cotabato, Philippines: Alamada, Aleosan, Antipas, Arakan, Banisilan, Carmen, Kabacan, Kidapawan City, Libungan, Magpet, Makilala, Matalam, Midsayap, Mlang, Pigcawayan, Pikit, President Roxas, and Tulunan. The rice production locations of these municipalities are shown in Fig. 1. Fig. 2 shows the differences of the rice production per municipality where in, in Fig. 2a, the municipality of Mlang amazingly dominates the overall production while the mountainous area of Banisilan is the lowest. Fig. 2b, and c show the irrigated and the rainfed rice production by municipality. As seen in Fig. 2b, Mlang topped the overall production in irrigated rice production and is second in the rainfed rice production. The municipality of Matalam got the highest rice production as shown in Fig. 2c.

In Fig. 2, you can observe the total rice production in Cotabato province, encompassing both irrigated and rainfed regions. Fig. 2a highlights the remarkable performance of the Municipality of Mlang, contributing a significant 25% share to the total rice production over a 15-year period. This achievement stands in stark contrast to Magpet, which recorded the lowest production figures.

Transitioning to Fig. 2b, we observe a graphical representation elucidating irrigated rice production dynamics within the provincial boundaries. Notably, the municipality of Aleosan emerges as a conspicuous outlier, exhibiting the least prolific rice cultivation performance. This phenomenon can be primarily attributed to Aleosan's topographical characteristics, characterized by its elevated terrain, rendering the implementation of irrigation infrastructure a formidable challenge. A similar predicament befalls several other upland municipalities in the vicinity, including but not limited to Alamada, Banisilan, Arakan, Antipas, Magpet, Kidapawan, and Makilala.

**Fig. 3:**
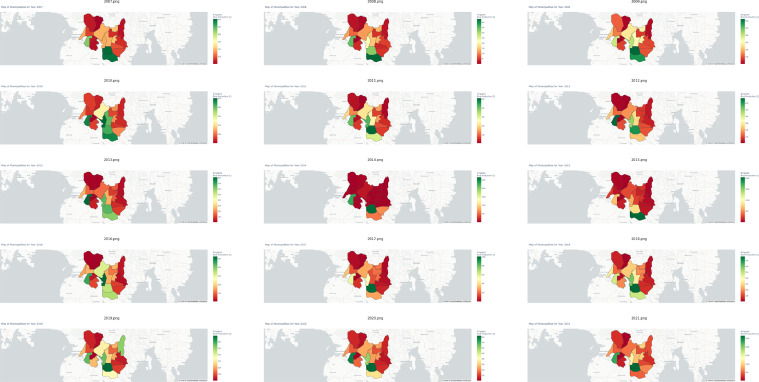
Yearly variations in irrigated rice production for each municipality in Cotabato from 2007 to 2021.

**Fig. 4:**
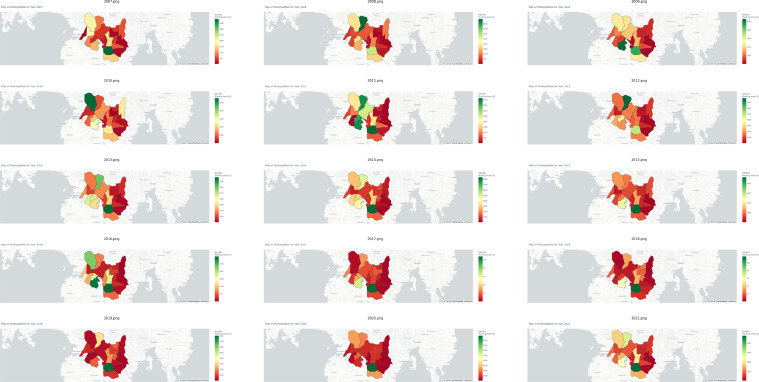
Yearly variations in rainfed rice production for each municipality in Cotabato from 2007 to 2021.

In stark contrast, the municipality of Mlang emerges as a preeminent exemplar of proficient irrigated rice production, boasting a substantial contribution of 17.08% to the aggregate output within the purview of Cotabato. Moreover, Fig. 2c delves into the realm of rainfed rice production, a domain typically characterized by relatively diminished yields. This phenomenon is largely attributable to the geographical disposition of Cotabato province, the majority of which encompasses lowlands conducive to paddy agriculture. Notably, Magpet, a municipality characterized by the lowest rainfed production output, deviates from the regional norm by specializing in the cultivation of alternative crops, such as rubber and corn. Within the sphere of rainfed rice production, the municipality of Mlang emerges as a paramount leader, commanding a remarkable 30.47% share of the total rainfed production amalgam. Subsequently, Fig. 3 delineates the annual spatiotemporal fluctuations in irrigated rice production, elucidating the intricate nuances within each municipality of Cotabato, spanning the chronological period from 2007 to 2021. A parallel representation is presented in Fig. 4, wherein analogous annual manifestations elucidate the spatial variances in rainfed rice production across the municipalities of Cotabato over the same temporal expanse, encompassing the years 2007 to 2021. For a comprehensive encapsulation of pertinent statistical information, please see Table 2.

**Table 2:** Summary statistics of irrigated and rainfed rice production during 2007-2021.

Municipality	Irrigated		Rainfed	
	Mean	Range	Mean	Range
Alamada	3,730.18	627.8–11,460.34	3,730.18	627.8–11,460.34
Aleosan	662.75	70–2,665.5	662.75	70–2,665.5.0
Antipas	10,096.39	1062–20,472.06	10,096.39	1062–20,472.06
Arakan	6,484.01	424.2–5,5145	6,484.01	424.2–5,5145.00
Banisilan	993.51	207.54–3,349.3	993.51	207.54–3,349.30
Carmen	22,984.99	11,825–31,738	22,984.99	11,825–31,738.00
Kabacan	44,817.00	45,26.4–66,039.79	44,817.00	4,526.4–66,039.79
Kidapawan City	6,654.29	154.81–10,213.47	6,654.29	154.81–10,213.47
Libungan	11,321.58	240.75–16,906.38	11,321.58	240.75–16,906.38
Magpet	4,420.73	485.84–8,780.87	4,420.73	485.84–8,780.87
Makilala	8,233.38	4,476–12,160.6	8,233.38	4,476–12,160.60
Matalam	22,858.61	336.75–40,759.3	22,858.61	336.75–40,759.3
Midsayap	53,685.31	34,982–81,255.88	53,685.31	34,982–81,255.88
Mlang	62,234.44	32,254–100,720	62,234.44	3,2254–10,0720.00
Pigcawayan	20,565.12	16,65.9–34,863.02	20,565.12	1,665.9–34,863.02
Pikit	9,499.13	2,181.5–22,875	9,499.13	2,181.5–22,875.00
President Roxas	16,967.07	3,047.9–28,934.36	16,967.07	3,047.9–28,934.36
Tulunan	43,472.68	23,683.81–60,889.42	43,472.68	23,683.81–60,889.42

**Fig. 5:**
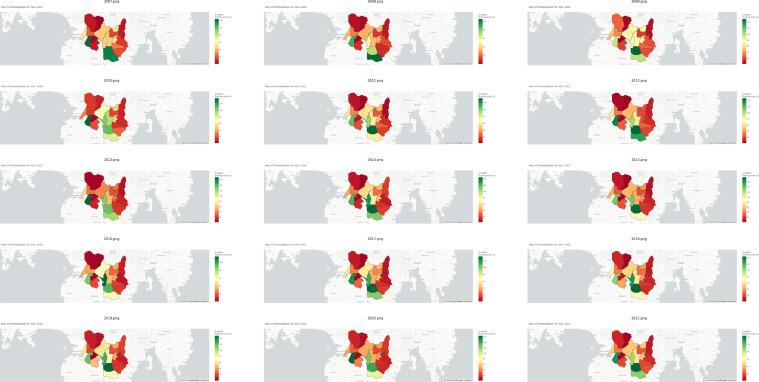
Yearly variations in irrigated cultivation area for each municipality in Cotabato from 2007 to 2021.

**Fig. 6:**
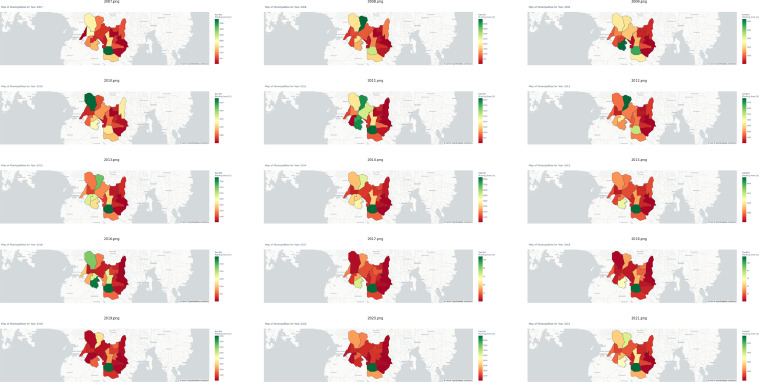
Yearly variations in rainfed cultivation area for each municipality in Cotabato from 2007 to 2021.

Figs. 5 and 6 provide valuable spatial data regarding irrigated and rainfed rice cultivation across different municipalities in the region for fifteen (15) years. These illustrate the distribution and relative significance of planting areas within each municipality in terms of irrigation and rainfed cultivation methods.

For irrigated rice cultivation area percentages, municipalities like Kabacan, Midsayap, and Mlang consistently exhibit high percentages, indicating their prominence in this aspect. These areas contribute significantly to the overall irrigated rice production landscape within the region. Conversely, concerning rainfed rice cultivation area percentages, municipalities such as Banisilan, Pikit, and Carmen demonstrate strong performance. These regions consistently achieve substantial rainfed rice production percentages, underscoring their proficiency in this cultivation method. This spatial data provides crucial insights into the geographical variations of irrigated and rainfed rice production percentages across municipalities, highlighting key contributors and their roles in the regional rice production landscape. Please see Table 3 for the summary statistics of cultivation area in Cotabato from 2007 to 2021.

**Table 3:** Summary statistics of irrigated and rainfed cultivation area of Cotabato during 2007-2021.

MUNICIPALITY	Irrigated		Rainfed	
	Mean	Range	Mean	Range
Alamada	942.87	261-2800	1,949.80	166-5526
Aleosan	220.73	28-799	2,103.80	76-4473
Antipas	2,310.13	1189-3219	720.47	24-2131
Arakan	979.87	434-1887	614.33	5-2441
Banisilan	252.67	46-913	3,204.33	668-7725
Carmen	5,366.47	3222-6985	1,210.00	182-2557
Kabacan	10,606.67	4684-13589	554.33	161-1324
Kidapawan City	1,903.80	944-2828	550.67	209-832
Libungan	2,924.27	1522-3741	758.80	172-1975
Magpet	1,266.07	622-2359	58.87	7-205
Makilala	1,770.33	989-2514	161.13	63-823
Matalam	5,745.27	3490-9129	1,975.00	971-3658
Midsayap	12,249.33	8785-16983	1,285.40	30-4536
Mlang	13,222.33	7078-17803	6,455.07	2342-11626
Pigcawayan	5,382.00	3083-7726	1,095.73	38-2495
Pikit	2,373.87	720-5079	3,297.60	842-6451
President Roxas	4,348.93	2283-6965	807.60	223-2503
Tulunan	9,265.13	5538-12897	1,514.13	

### 2.2 Agro-Climate Data

**Fig. 7:**
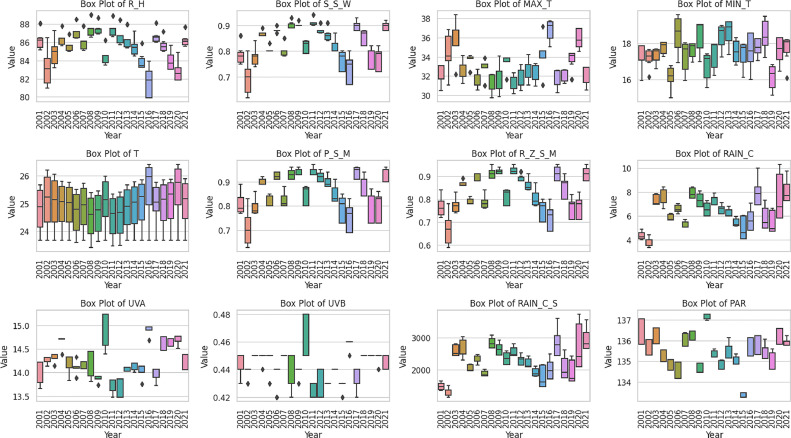
Generated yearly box plots depicting agro-climate parameters in Cotabato, organized and labeled according to their respective nomenclature.

Various papers also discussed the effect climate change on rice [4], [5], [6], [7], [8], [9], [10]. The presented data encompasses various agro-climate parameters specific to a given region. The target agro-climate factors used in this study are listed in Table 4. Under the Humidity category, we have two sub-variables: “Relative” representing relative humidity measured in percentages, and “Specific'' indicating specific humidity, also expressed as a percentage. Rainfall includes “Precipitation Corrected,” which measures corrected rainfall in millimeters (mm), and “Precipitation Corrected Sum,” which represents the cumulative corrected rainfall in mm. In the Temperature section, we find “Maximum” and “Minimum,” recording the highest and lowest daily temperatures in degrees Celsius (°C), respectively. Under Radiation, we have “All Sky Surface UVA Irradiation” and “UVB Irradiation,” quantifying UVA and UVB radiation in Watts per square meter (W/m^2), as well as “Clear Sky Surface PAR Total,” measuring total photosynthetically active radiation under clear sky conditions in W/m^2. Lastly, Soil Moisture consists of “Surface Soil Wetness,” denoting the moisture content of the topsoil in grams of water per gram of soil (g/g), “Profile Soil Moisture” measured in volumetric water content (VWC), and “Root Zone Soil Wetness,” also expressed in VWC. This dataset, sourced from NASA's POWER project, underwent rigorous data processing to calculate the annual means for each variable by municipality and year. This extensive data processing was performed to present the information in a more comprehensible and succinct manner. Detailed information regarding this data processing, including quality control procedures and any adjustments made, can be found in this article [1], and is complemented by box plots in Fig. 7 for each parameter on a yearly basis. These box plots are instrumental in visualizing the agro-climate data, aiding in the understanding of their patterns and variations. It is specifically employed for predicting variations in rice production within Cotabato Province, providing valuable insights into the environmental conditions, including humidity, rainfall, temperature, radiation, and soil moisture, crucial for agro-climate analysis and research.

**Table 4:** Agro-climate data variables and descriptions.

Variable	Sub-Variable	Description	Nomenclature	Unit
Humidity	Relative	Relative humidity expressed as a percentage.	R_H	%
	Specific	Specific humidity expressed as a percentage.	S_H	%
Rainfall	Precipitation Corrected	Precipitation corrected rainfall measured in mm.	Rain_C	mm
	Precipitation Corrected Sum	Cumulative precipitation corrected rainfall measured in mm.	Rain_C_S	mm
Temperature	Maximum	Maximum daily temperature in degrees Celsius.	MAX_T	°C
	Minimum	Minimum daily temperature in degrees Celsius.	MIN_T	°C
Radiation	All Sky Surface UVA Irradiation	All sky surface UVA irradiation in Watts per square meter (W/m^2).	UVA	W/m^2
	All Sky Surface UVB Irradiation	All sky surface UVB irradiation in Watts per square meter (W/m^2).	UVB	W/m^2
	Clear Sky Surface PAR Total	Clear sky surface PAR total irradiation in Watts per square meter (W/m^2).	PAR	W/m^2
Soil Moisture	Surface Soil Wetness	Surface soil wetness expressed as grams of water per gram of soil (g/g).	S_S_W	g/g
	Profile Soil Moisture	Profile soil moisture measured in volumetric water content (VWC).	P_S_M	VWC
	Root Zone Soil Wetness	Root zone soil wetness measured in volumetric water content (VWC).	R_Z_S_W	VWC

## 3 Experimental Design, Materials and Methods

### 3.1 Data Collection

The data collection process involved gathering data from multiple sources, including online agro-climate datasets from NASA's POWER project and statistics on rice production, and producing areas provided by the Cotabato OPAG. The data was downloaded by the municipality of Cotabato and classified by year and municipality. The quality of the data was confirmed by inspecting the structure of the table, and unnecessary variables were removed while the required variables were prioritized.

### 3.2 Data Preparation

The Extract Transform and Loading (ETL) process was conducted within Power BI tool, where the data was extracted, cleaned, and formatted before being loaded into a data warehouse for exploratory data analysis. The extraction process focused on selecting and preparing the final data collection, while the transformation process involved integrating data and constructing aggregates.

### 3.3 Data Analysis

The exploratory data analysis involved iteratively asking a series of questions about the data and trying to build hypotheses based on the insights gained from the data. Regression analysis was employed to statistically support the relevance of the collected agro-climatic historical data as explanatory variables that have significant relevance over rice yield historical data of Cotabato as the response variable using the Power BI and Python Script. Multivariate linear regression was used to assess the strength of the relationship between variables and model the future between them. The multivariate linear regression formal is given as [11]:

(1) Y=b0+b1x1+b2x2+⋯+bnxn

Where:

*b*0: Intercept

*b*1…*bn*: Slope coefficients for each explanatory variable

*x*1…*xn*: explanatory variables

## Limitations

The dataset under examination presents a valuable resource for researchers, policymakers, and analysts interested in understanding the complex interplay between agro-climate conditions and rice production in Cotabato Province, Philippines. This dataset encompasses two critical components: crop-related data and agro-climate data, spanning a timeframe from 2007 to 2021. It offers a window into the challenges and opportunities facing rice cultivation in the region, making it an indispensable asset for various fields, including agriculture, climate science, and policymaking.

However, as with any dataset, it is essential to consider its inherent limitations to ensure that analyses and conclusions drawn from it are well-founded and informed. In this discussion, we explore four primary limitations associated with this dataset. These limitations encompass the temporal scope, data quality, and the necessity to account for spatial variation within the province.

## Ethics Statement

This work did not involve studies with animals and humans. Also, we acquired explicit permissions to access and utilize the primary data from the Provincial Agriculturist office of Cotabato. These permissions enabled us to responsibly harness this valuable source while upholding ethical and legal standards.

## CRediT authorship contribution statement

**Reymark D. Delena:** Conceptualization, Methodology, Software, Data curation, Writing – original draft, Visualization. **Marivic S. Tangkeko:** Conceptualization, Methodology, Data curation, Writing – review & editing, Supervision. **Joseph C. Sieras:** Conceptualization, Methodology, Writing – review & editing, Funding acquisition.

## Data Availability

Dataset on Rice Yield in Cotabato Province (Original data) (Mendeley Data).
